# Clinicopathologic characteristics and prognosis of upper tract urothelial carcinoma complicated with aristolochic acid nephropathy after radical nephroureterectomy

**DOI:** 10.1186/s12906-020-2861-5

**Published:** 2020-06-03

**Authors:** Hongli Shan, Wen Tian, Yazhao Hong, Bo Xu, Chunxi Wang, Bing Yu, Xiaoqing Wang

**Affiliations:** 1 Department of clinical laboratory, The First Hospital of Jilin University, Changchun, 130021 People’s Republic of China; 2 Department of Blood Transfusion, The Second Hospital of Jinlin University, Changchun, 131000 People’s Republic of China; 3 Department of urology, The First Hospital of Jilin University, Changchun, 130021 People’s Republic of China

**Keywords:** Upper tract urothelial carcinoma, Aristolochic acid, Carcinogenesis, Nephroureterectomy, Survival

## Abstract

**Background:**

The purpose of this study was to identify the clinicopathologic characteristics and prognosis of upper tract urothelial carcinoma (UTUC) patients complicated with aristolochic acid nephropathy(AAN) after radical nephroureterectomy (RNU).

**Methods:**

The clinical data of 42 UTUC patients with AAN (AAN group) and 238 UTUC patients without AAN (Non-AAN group) were retrospectively reviewed. All patients received a RNU with excision of bladder cuff. Demographic and clinical data, including preoperative indexes, intraoperative indexes and surgical outcomes were compared.

**Results:**

There were no significant differences in age, tumor location, surgery approach, tumor pathologic grade, stage, the mean operative time and estimated blood loss between the two groups (all *p* > 0.05). There were more female patients in the AAN group (*p* < 0.001), and 57.1% were high grade tumors. The AAN group showed a higher complications rate (*p* = 0.003). The median follow-up time was 43.2 months. The AAN group showed a worse estimated 5-year overall survival rate (35.1% vs. 63.0%, *p* = 0.014), however, no significant difference was found between the two groups with regard to disease specific survival (63.5% vs. 81.5%, *p* = 0.091). Multivariate binary logistic regression analysis showed that AAN was an independent factor related with overall and disease specific survival. 38.9% of all patients experienced any types of recurrence, and the estimated 5-year recurrence-free survival rate was lower in the AAN group (37.1% vs. 63.7%, *p* = 0.001). In the comparison of subgroups stratified by recurrence type, the AAN group had a higher intravesical (*p* = 0.030) and contralateral recurrence rate (*p* = 0.040).

**Conclusion:**

UTUC with AAN occurred more frequently in female patients who were more likely to develop high-grade tumors. However, these patients showed a worse overall survival and a lower recurrence-free survival rate than the other patients. AA-related UTUC might be associate with an increased risk of intravesical and contralateral recurrence after RUN.

## Background

Upper urinary tract urothelial cell carcinoma (UTUC) represents a relatively rare tumor entity and account for 5–10% of all urothelial carcinomas [1]. The incidence rate has risen worldwide over the past few decades, partly as a result of improvements in UTUC detection and survival [2, 3]. According to the recent EAU Guidelines, several studies have demonstrated a carcinogenic potential of aristolochic acid (AA) contained in Aristolochia fangchi and Aristolochia clematis [1, 4, 5]. Aristolochic acid is implicated in multiple cancer types, sometimes with very high mutational burdens, especially in UTUCs [6].

Chinese herbal medicine is an important part of traditional Chinese medicine, and it is considered to be an effective alternative treatment all over the world [7]. However, some of these drugs contain nephrotoxins and mutagens in the form of AAs and similar compounds [7, 8]. It causes extensive interstitial fibrosis and severe loss of renal tubules, termed aristolochic acid nephropathy (AAN). UTUC usually occurs after the diagnosis of AAN. AA-associated AAN and UTUCs are prevalent in Taiwan [4, 9]. A meta-analysis established an odds ratio of 5.97 for developing UTUC after exposure to AA, and this risk may persist for many years (sometimes> 10 years) after stopping exposure to AA [10].

Compared to UTUC patients without AAN, the oncologic results of UTUC patients with AAN after RNU are unsure. There are only a few reports in the literature about the prognosis of the disease. Cukuranovic et, al. retrospectively analyzed the data of UTUC patients in the Balkans, the results showed that the Balkan endemic nephropathy patients were more likely to develop lower stage and lower grade UTUC. However, the disease specific survival was the same in the Balkan endemic nephropathy patients and control settlements [11]. Other reports indicated that AA exposure was associated with worse disease specific survival, higher intravesical and contralateral recurrence rate [9, 12]. This study was designed to investigate the clinicopathologic characteristics and oncologic outcomes of UTUC patients with AAN after RNU.

## Methods

### Clinical data

The data of patients diagnosed with UTUC with or without AAN who had undergone RNU and bladder cuff resection on-site were collected from the electronic patient database of the First Hospital of Jilin University. This study was approved by the Ethics Committee of the First Hospital of Jilin University, and written informed consent from patients was obtained. AAN was diagnosed according to previous research: (1) the presence of a definite history of taking AA-containing medications prior to disease onset; (2) the presence of obvious tubular dysfunction, with or without diminished GFR; (3) the absence of recent or long-term ingestion of antibiotics, non-steroidal anti-inflammatory drugs, diuretics or Chinese traditional medicines containing minerals or metals and (4) the absence of evidence of other glomerular or tubulointerstitial diseases caused by infectious or immune diseases; (5) Tubulointerstitial nephropathy was pathologically confirmed by renal biopsy or postoperative specimen examination [13, 14]. From January 2010 to January 2017, electronic records of 42 UTUC patients with AAN (AAN group) and 238 UTUC patients without AAN (non-AAN group) were retrospectively assessed for the recent study.

The UTUC patients were diagnosed by computed tomography (CT) urography, intravenous urography, urinary cytology or ureteroscopy with or without biopsy. Cystoscopies and CT scan were performed to rule out a concomitant bladder cancer and distal metastases. Radical nephroureterectomies were performed by laparoscopic or open strategy, whereby the bladder cuff resections were performed by open extravesical approach in all cases. A single dose of intravesical mitomycin C was given to all patients after the surgery to prevent bladder recurrence. Selected patients who had an advanced disease confirmed by preoperative imaging or postoperative pathology received a neoadjuvant (gemcitabine and cisplatin) or adjuvant chemotherapy (gemcitabine and cisplatin).

### Follow-up

Cystoscopy and urinary cytology was carried out once every 3 months for the first year, and every 6 months thereafter until 5 years. CT urography was performed every 6 months over 2 years and then yearly. A bone scan, chest CT, and magnetic resonance imaging were performed, if necessary. Disease recurrence was defined as local or contralateral recurrence, intravesical recurrence and distant metastasis.

### Statistical analysis

Data are expressed as absolute numbers and percentages, means with SD, or as medians with interquartile ranges as appropriate. IBM SPSS statistics version 20.0 was used for all analyses. For all statistical tests, *P* < 0.05 was considered to indicate a significant difference. Chi-square test or Fisher’s exact test was used to categorical data and unpaired *t* test and Mann-Whitney *U* test for continuous data with and without a normal distribution, respectively. The Kaplan-Meier method was used to calculate survival rates. Multivariate binary logistic regression was used to evaluate if AAN was associated independently with survival from other factors. Recurrence was evaluated from the date of surgery. Tumor recurrence-free survival was defined as the interval from surgery to the first appearance of intravesical, local, or contralateral recurrence, and distant metastasis to the end of the study, whichever came first.

## Results

No statistically significant differences were found regarding patients’ age, tumor site, surgical approach, pathologic tumor stage and grade between the two groups. There were more female patients in AAN group (*p* < 0.001), whereby 57.1% of patients in this group had high grade tumours. End-stage renal disease was equally more common in the AAN group than in non-AAN group (*p* < 0.001). No significant differences in terms of operative time and estimated blood loss were observed between the two groups (142.3.3 ± 26.4 min vs. 146 ± 32.5 min, 207.8 ± 78.3 ml vs. 194.5 ± 90 ml, *p* > 0.05, respectively). Complications included intraoperative bleeding, spleen injury, pleural injury, fever, deep venous thrombosis, incision infection, as well as cardiovascular and cerebrovascular complications. Three patients had a cardiovascular complication and required an intensive care unit stay. According to Clavien-Dindo grading system, the distribution of complications was higher in AAN group (*p* = 0.003) than in non-AAN group of patients (Table 1).

**Table 1:** Patient characteristics of the two groups

Variables	AAN(*n* = 42)	NON-AAN(*n* = 238)	*P* Value
Age (year)	68.0 (52–81)	67.2 (32–79)	0.62
Gender, n (%)			< 0.001
Male	11 (26.2)	142 (59.7)	
Female	31 (73.8)	96 (40.3)	
End-stage renal disease			< 0.001
Yes	14 (33.3)	11 (4.6)	
No	28 (66.7)	227 (95.4)	
Tumor location, n (%)			0.89
Renal pelvis	23 (54.7)	112 (62.7)	
Ureter	19 (45.3)	126 (37.3)	
Upper	4 (23.1)	36 (29.0)	
Middle	9 (46.2)	60 (47.4)	
Lower	6 (30.7)	30 (23.6)	
Previous bladder tumor n (%)			0.40
Yes	5 (11.9)	21 (9.3)	
No	37 (88.1)	217 (91.7)	
Hydronephrosis			
Yes	27 (64.3)	156 (65.5)	0.87
No	15 (35.7)	82 (34.5)	
pT stage, n (%)			0.79
pTa	1 (2.4)	7 (2.8)	
pT1	19 (45.2)	109 (45.8)	
pT2	15 (35.7)	86 (36.1)	
pT3	4 (9.5)	26 (11.1)	
pT4	3 (7.2)	10 (4.2)	
pN stage, n (%)			0.12
pN0	38 (90.7)	205 (86.1)	
pN1	4 (9.3)	33 (13.9)	
Grade, n (%)			0.11
Low	18 (42.8)	102 (43.1)	
High	24 (57.1)	136 (56.9)	
Surgical approach, n (%)			0.40
Laparoscopic	36 (85.7)	212 (89.1)	
Open	6 (14.3)	26 (10.9)	
Mean operative time (min)	142.3 ± 26.4	146.0 ± 32.5	0.31
Mean estimate blood loss (ml)	207.8 ± 78.3	194.5 ± 90.0	0.06
Complications (Clavien Grade), n (%)			0.003
None	20 (47.6)	163 (68.5)	
1	11 (26.2)	43 (18.1)	
2	7 (16.7)	28 (11.8)	
3	1 (2.4)	3 (1.3)	
4	3 (7.1)	1 (0.4)	
5	0 (0)	0 (0)	

**Fig. 1:**
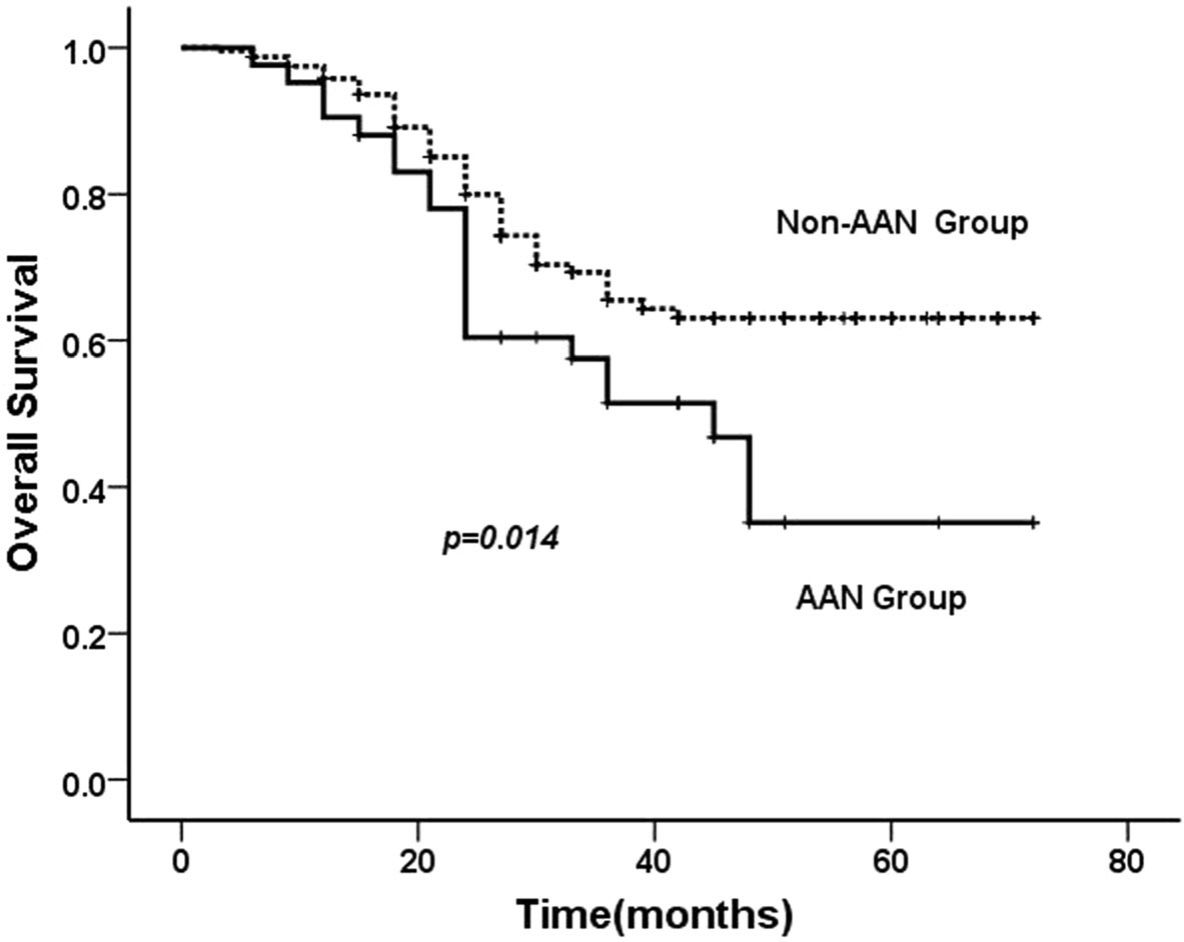
Cumulative incidence of overall survival following radical nephroureterectomy between AAN and Non-AAN group

**Fig. 2:**
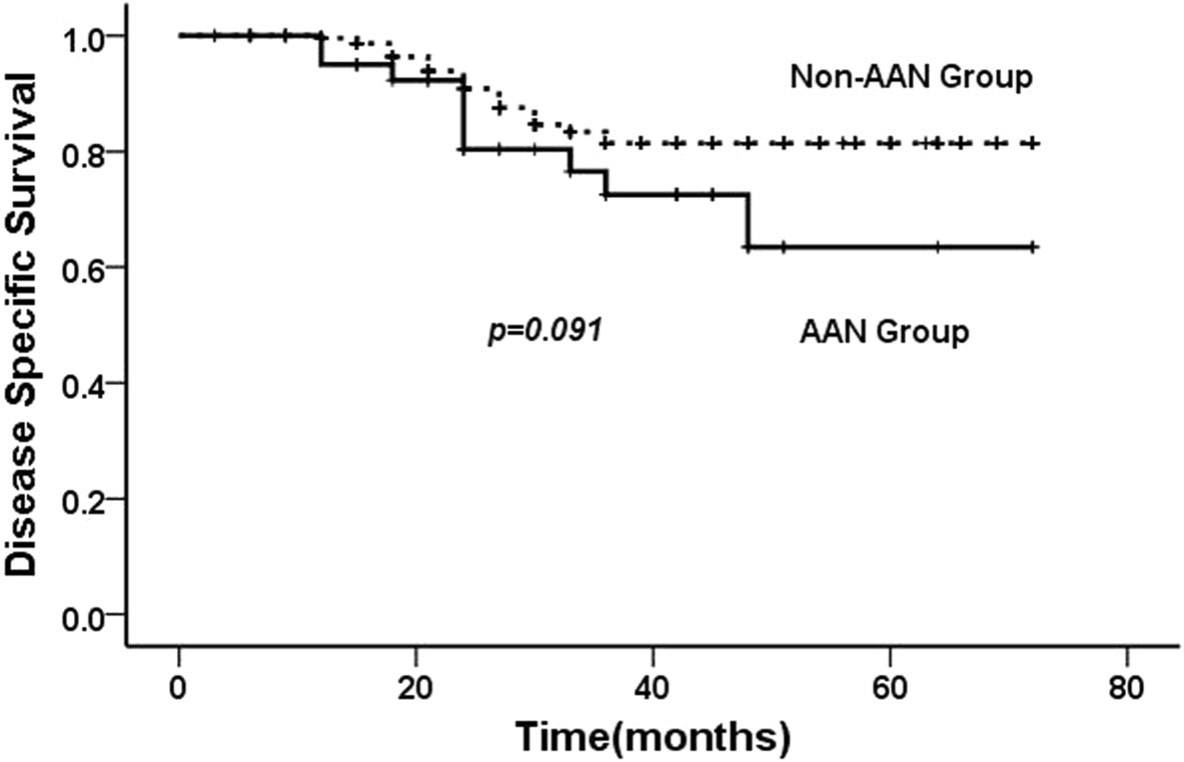
Cumulative incidence of disease specific survival following radical nephroureterectomy between AAN and Non-AAN group

Mean follow-up period was 43.2 (range, 6–72) months. In the AAN group, 10 patients died of metastatic disease and 12 died of other disease. The estimated 5-year overall survival rate and the estimated 5-year disease specific survival rate were 35.1 and 63.5%, respectively. In the Non-AAN group, 35 patients died of UTUC and 43 patients died of other causes. The estimated 5-year overall survival rate and the disease specific survival rate were 63.0 and 81.5%, respectively. The AAN group showed a worse overall survival (*p* = 0.014) (Fig. 1), however, there was no significant difference between the two groups with regard to disease specific survival (*p* = 0.091) (Fig. 2). Multivariate binary logistic regression analysis showed that AAN, T stage, grade, and positive lymph node were the independent factors related with overall and disease specific survival (Table 2).

**Table 2:** The risk factors related with the overall and disease specific survival

Variables	Overall Survival		Disease Specific Survival	
	*P* value	HR(95% CI)	*P* value	HR(95% CI)
Age (years)	0.483	1.012 (0.978 to 1.047)	0.062	1.053 (0.899 to 1.111)
Stage(T2-T4 vs Ta,T1)	0.019	2.048 (1.637 to 3.493)	0.005	3.226 (1.426 to 7.297)
Lymph node(N1 vs N0)	< 0.001	9.417 (2.849 to 18.461)	0.003	4.860 (1.718 to 13.751)
Grade (high vs low)	0.014	2.041 (1.159 to 3.597)	0.027	2.745 (1.123 to 6.712)
AAN (yes vs no)	0.020	2.370 (1.428 to 4.902)	0.020	3.061 (1.190 to 7.872)

**Fig. 3:**
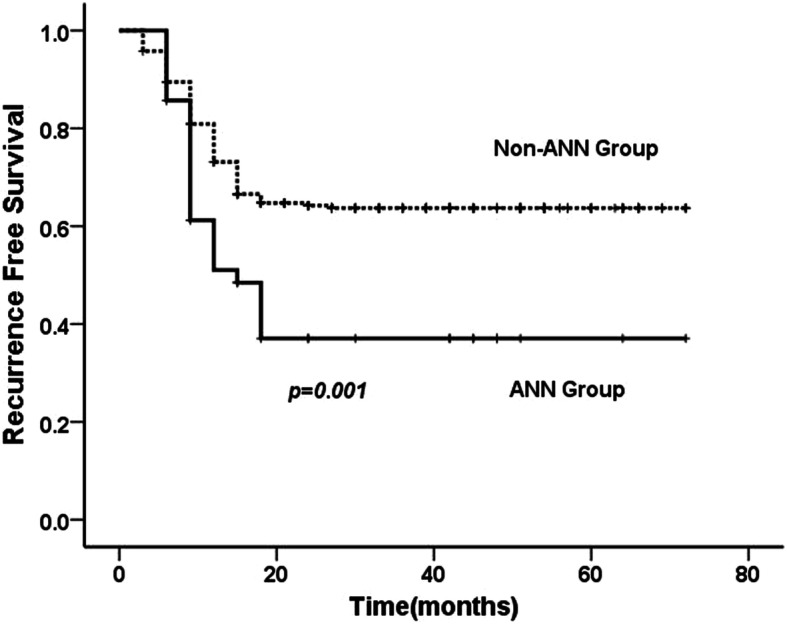
Kaplan-Meier estimates of recurrence-free probabilities following radical nephroureterectomy between AAN and Non-AAN group

**Fig. 4:**
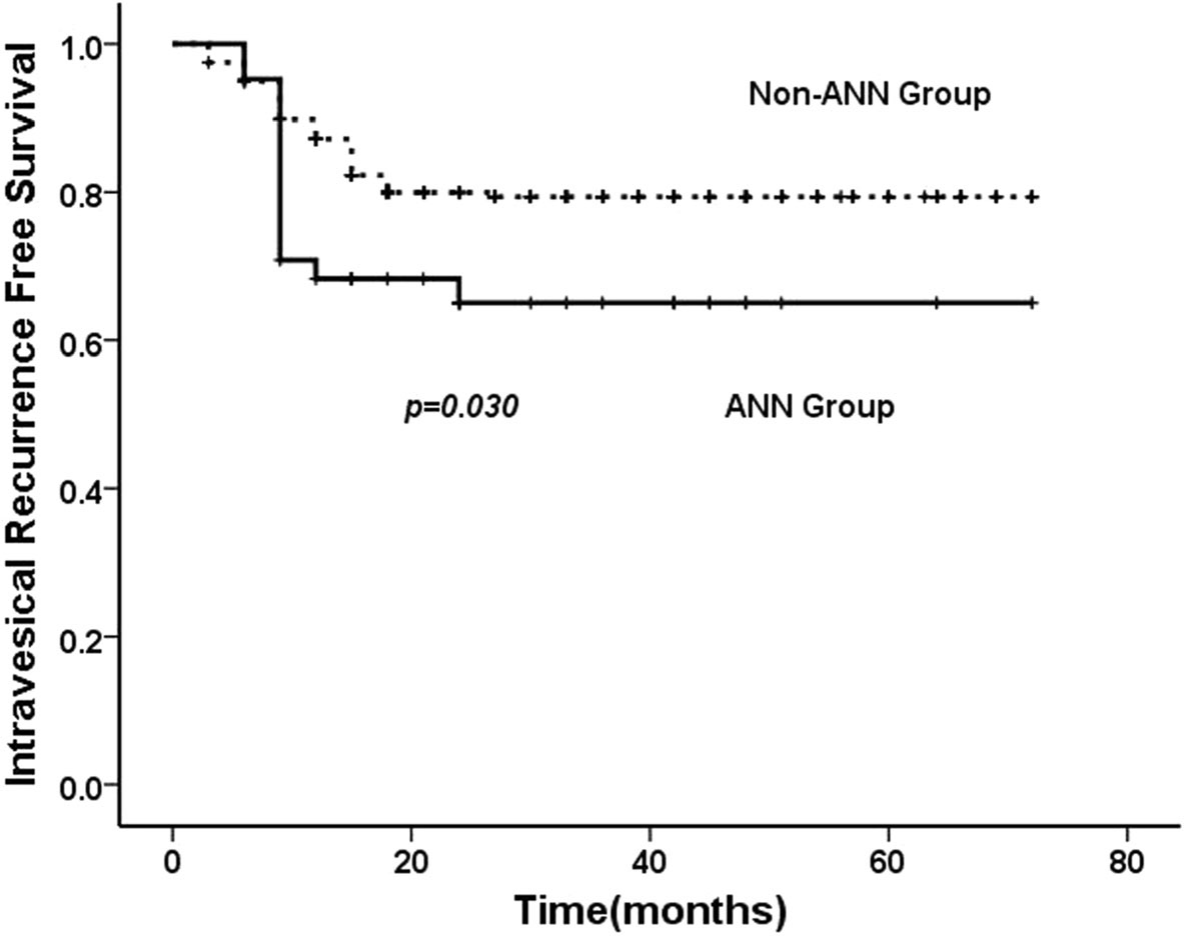
Kaplan-Meier estimates of intravesical recurrence-free probabilities following radical nephroureterectomy between AAN and Non-AAN group

Three types of tumor recurrence for UTUC were assessed, namely local, intravesical, as well as contralateral. No distant metastases was found before local recurrence. 38.9% patients experienced any type of recurrence during the follow up. Intravesical recurrence occurred in 61 (AAN, *n* = 14; non-AAN, *n* = 47), local recurrence in 30 (AAN, *n* = 5; non-AAN, *n* = 25) and contralateral recurrence in 18 (AAN, *n* = 6; non-AAN, *n* = 12) patients, respectively. The estimated 5-year recurrence-free survival rate in the AAN group was 37.1 and 63.7% in the non-AAN group, the recurrence-free survival probabilities differed significantly between the two groups of patients (*p* = 0.001)(Fig. 3). In the comparison of subgroups stratified by recurrence type, the AAN group had a higher estimated 5-year intravesical recurrence-free survival rate (65.0% vs 79.4%, *p* = 0.03) (Fig. 4) and contralateral recurrence rate (*p* = 0.040).

## Discussion

Aristolochic acid represents a carcinogenic, mutagenic and nephrotoxic compound commonly present in members of the plant family Aristolochiaceae and is widely used in Chinese herbs, dietary supplements, slimming pills, and contaminated flour [15–17]. Many studies have shown that exposure to AA is involved in the genesis of the UTUC [9, 12, 18] and other cancers [6, 19]; however, its tumorigenic role has yet to be understood. Currently, the main carcinogenic mechanism of AA is as follows: A:T to T:A transversions occurring in the 5′-CpApG-3′ trinucleotide context of the TP53 gene is considered to be the signature mutation of AA [15, 20]. Genes including H-ras, FGFR3, N-ras and BRCA2 are also involved, particularly in UTUC [16, 21, 22]. To elucidate the role of AA in tumorigenesis, the molecular signature of AA needs to be explored.

The highest incidence of AA-related UTUC worldwide is recorded in Asia [7, 9, 12], with the tumors occurring more frequent in females than males. In our cohort study, the AAN group consists of more female than male patients, and most of these female patients have a history of exposure to AA-related drugs (specifically to treat coronary heart disease and hepatitis) for more than a decade. AAN usually occurs before UTUC, and 14 patients in this study developed UTUC during dialysis. The most common symptoms of UTUC are visible or nonvisible hematuria, flank pain and system syndrome (including anorexia, weight loss, malaise, fatigue, fever, night sweats, or cough). Moreover, hydronephrosis is accidentally detected in patients undergoing health examination for different reasons [1]. For patients with AAN, the possibility of UTUC should be considered when they have hematuria and hydronephrosis. CT urography exhibits the highest diagnostic accuracy of available imaging techniques. In patients with AAN, magnetic resonance imaging is an option when CT urography is not considered because of the renal function. Diagnostic ureteroscopy is used for visualization of the ureter, renal pelvis, and collecting system, as well as for biopsy of suspicious lesions. The procedure is particularly useful in patients with diagnostic uncertainty. Multifocal tumors of AAN-related UTUC may occur; thus, contralateral ureteroscopy is also recommended to rule out lesions.

Radical nephroureterectomy with bladder cuff excision is the standard treatment for UTUC, regardless of surgery approach (open or laparoscopic) [23–26]. Seisen et al. recommended kidney-sparing management as a treatment option for patients with low-risk tumors [27, 28]. In the current study, patients who underwent kidney-sparing surgery were not enrolled because of the limited sample size. Nortier et al. performed prophylactic removal of native kidneys and ureters in 39 patients with end-stage AAN who were being treated with either transplantation or dialysis. These patients neither manifested clinical symptoms such as hematuria nor exhibited positive findings in preoperative imaging studies. Histological examination of surgical specimens revealed 18 cases of urothelial carcinoma, 19 cases of mild-to-moderate urothelial dysplasia, and 2 cases of normal urothelium. Prophylactic RNU with bladder cuff excision was recommended for patients with end-stage AAN, particularly those with unilateral UTUC [29]. Postoperative intravesical chemotherapy significantly decreases the risk of bladder recurrence after RNU for UTUC [1, 30]. However, no oncological results regarding intravesical chemotherapy in UTUC patients with AAN have been reported.

One study showed that patients with AAN were more likely to develop lower-stage and lower-grade UTUC in the Balkans, suggesting the reduced potential for malignancy [11]. However, in Taiwan, studies indicated that AA-related UTUC is highly aggressive and typically diagnosed as high-grade and high-stage carcinoma; recurrence in the contralateral upper urinary tract is also likely [9, 21]. Our cohort study showed that 57.1% of patients with AA-related UTUC had high grade tumors, similar to patients without AAN. The comparison of the estimated 5-year cancer specific survival rate between the two groups showed no significant difference. Thus, exposure to AA may not associated with worse disease specific survival, and the results vary from those of previously published studies [12].

The estimated 5-year overall survival rate was slightly lower, and death from other diseases was higher in the AAN group. Patients with AAN, particularly those undergoing maintenance hemodialysis, might also have various cardiovascular and cerebrovascular diseases related to chronic renal insufficiency. Studies showed that the cardiovascular mortality is 10–20 fold greater in patients receiving dialysis, relative to that in age and sex-matched controls without chronic kidney disease. Anemia, disordered bone mineral metabolism and oxidative stress also contribute to poor cardiovascular outcomes [31, 32]. Studies have shown that the risk of hospitalized stroke is significantly higher in patients receiving dialysis than in the general population [33].

Some studies have indicated that AA-related UTUCs are highly aggressive and that relapse may occur in the contralateral upper urinary tract [9, 12]. Our results showed that the estimated 5-year recurrence-free survival rate was significantly lower, and the intravesical and contralateral recurrence rate was higher in the AAN group than in the non-AAN group. These results may be attributed to the following: (1) AA related UTUC may be a multifocal disease. Tumors may occurs simultaneously or sequentially in any part of the urinary tract. Nortier et al. found multifocal tumors in end-stage AAN patients who had received a prophylactic removal of the native kidneys and ureters [29]. Another study determined that tumor multifocality and native AAN are significant independent risk factors affecting the development of initial intravesical recurrence after surgery for primary UTUC [34]. (2) The carcinogenic effects of AA may last for many years even if the drug containing AA is discontinued. Jelakovic’s molecular epidemiologic study reported that AL-DNA adducts and TP53 mutational signature can persist for years after exposure to AA [35]. A higher risk of subsequent development of bladder urothelial carcinoma was also identified in kidney transplant recipients with AAN after bilateral RNU, and it may occur years after AA is discontinued [36]. Owing to the high intravesical and contralateral recurrence rate in patients with AA related UTUC, active follow-up should be conducted after RNU.

The current study has several limitations. First, the small sample sizes and the nature of the retrospective study design and may limit the generalizability of our results. Second, bias in patient enrollment may be present. The patients with a history of exposure to AA but had no AAN were not excluded in the non-AAN group, which can introduce selection bias to our final results. Gene sequencing should be done to identify whether these patients have A:T-to-T:A transversions in TP53. Moreover, we excluded the AA-related patients who received kidney-sparing surgery due to renal insufficiency; these patients might have exhibited more characteristics of AA-related UTUC. Further, the relatively short follow-up time prevented the observation of additional contralateral and intravesical recurrences as well the analysis as long-term cancer specific survival.

## Conclusion

UTUC with AAN occurred more frequently in female patients who were more likely to develop high grade tumors. They had the same estimated 5-year cancer-specific survival rate as that of UTUC patients without AAN. However, these patients showed a worse overall survival- and a lower recurrence-free survival rate than the other patients. AA-related UTUC might be associate with an increased risk of intravesical contralateral recurrence after RUN. Owing to the limitations of the study, well-designed prospective trials need to be conducted to confirm our findings.

## Data Availability

The datasets analyzed during the current study is available from the corresponding author on reasonable request.

## Abbreviations

AA: Aristolochic acid
AAN: Aristolochic acid nephropathy
CT: Computed tomography
RNU: Radical nephroureterectomy
UTUC: Upper tract urothelial carcinoma
